# Exploring the experiences of having Guillain‐Barré Syndrome: A qualitative interview study

**DOI:** 10.1111/hex.13116

**Published:** 2020-08-03

**Authors:** Joseph N. A. Akanuwe, Despina Laparidou, Ffion Curtis, Jennifer Jackson, Timothy L. Hodgson, Aloysius Niroshan Siriwardena

**Affiliations:** ^1^ Community and Health Research Unit (CaHRU) School of Health and Social Care University of Lincoln Lincoln UK; ^2^ Lincoln International Institute for Rural Health University of Lincoln Lincoln UK; ^3^ Lincoln International Business School University of Lincoln Lincoln UK; ^4^ School of Psychology University of Lincoln Lincoln UK

**Keywords:** experiences, Guillain‐Barré Syndrome, patients, recovery

## Abstract

**Background:**

Guillain‐Barré syndrome (GBS) is a rare inflammatory disorder affecting the peripheral nerves. Although typically there is full neurological recovery, some people continue to experience residual physical, psychological or social problems longer term. Evidence describing the experiences of people with GBS is limited.

**Objective:**

We aimed to explore the experiences of people with GBS in the UK.

**Design:**

We used qualitative (face‐to‐face and telephone) interviews to explore experiences of people with GBS. Audio‐recorded data were transcribed verbatim and analysed using the Framework Method supported by NVivo 11.

**Setting and Participants:**

We purposively recruited a sample of 16 volunteers with a prior diagnosis of GBS of varying age, sex, ethnicity, location, marital status, time since diagnosis and length of hospital stay to maximize differences in experience. Interviewees were required to have been discharged from hospital, able to give informed consent, able to speak and understand English and currently resident in the United Kingdom.

**Results:**

The key themes arising from the analysis were as follows: the importance of early diagnosis; the experiences of inpatient care; the importance of active support for recovery; the need for communication throughout the course of the illness; the need for greater awareness, knowledge and provision of information by health‐care staff; and path to achieving function.

**Conclusion:**

This is the first qualitative study exploring experiences of people with GBS in the UK through their whole illness journey from onset to recovery. The findings contribute to our understanding of the experiences and support needs of people recovering from GBS.

## 1 BACKGROUND

Guillain‐Barré syndrome (GBS) is a rare neurological disorder in which the body's immune system mistakenly attacks part of its peripheral nervous system following an acute infection or other immunological challenge.^1^ GBS is a monophasic post‐infectious condition, affecting peripheral nerves with variable presentation and severity, following which most people with the condition experience full neurological recovery and resolution. The incidence of GBS is approximately one in 100 000 per year and, while more common in adults and the elderly, it can also affect children and adolescents.^1^ There are variants of the condition, for example affecting the eye muscles (Miller‐Fisher syndrome), leading to motor but not sensory loss (acute motor axonal neuropathy [AMAN]), or causing distal weakness and sensory symptoms (acute motor and sensory axonal neuropathy [AMSAN]).^2^

The pathophysiology of GBS begins with acute inflammatory damage to the myelin sheath covering motor and sensory nerves or the nerve axons themselves, which causes subacute onset weakness and abnormal sensation.^2^ The inflammation damaging the nerves is short‐lived, and then, the nerves recover slowly as the damaged myelin or axon regenerates. The initial symptoms take different times to recover, or sometimes do not completely recover depending on the extent of the initial damage.^2^ Following recovery from the acute phase of the illness, incompletely recovered or scarred nerves can cause long‐term symptoms, and some people continue to experience these residual problems, either physical, psychological or social, months or even years later.^1^ Studies, mostly based on quantitative methods, have reported residual physical problems in 20%‐30% of patients,^3^, ^4^, ^5^ and long‐term changes in leisure and social activities in 27%‐37% of patients.^6^, ^7^ These residual problems have reportedly led to reduced quality of life for patients several years after the onset of the condition.^8^, ^9^

Few qualitative studies have previously been conducted exploring experiences of people with GBS. Two studies focused on experiences at the onset and during acute hospital care.^10^, ^11^ Further studies explored experiences of the recovery process from dependency to regaining independence,^12^ experiences of returning to work as a significant step in the recovery process^13^ and experiences of disability in everyday life and managing the recovery process after falling ill with GBS.^14^ With the exception of one study,^13^ previous studies were conducted outside the United Kingdom (UK). In this study, we aimed to use qualitative methods to explore the experiences of people with GBS in the UK.

## 2 METHODS

### 2.1 Design, sampling, identification and recruitment

We used a qualitative interview design with individuals previously diagnosed with GBS. The Illness Trajectory Framework (ITF)^15^ was used to inform data collection, analysis and reporting.

Illness Trajectory Framework argues that illnesses have a trajectory which involves the following sequential phases: (a) initial or pre‐trajectory phase (before any signs or symptoms); (b) trajectory onset phase (onset of signs and symptoms leading to diagnosis); (c) crisis phase (experience of a potentially life‐threatening situation); (d) acute phase (when symptoms can be controlled by a prescribed treatment); (e) stable phase (when symptoms are controlled); (f) unstable phase (when symptoms are not controlled despite the treatment regimen applied in the previous stage); (g) downward phase (progressive deterioration in mental and physical status); and (h) dying phase (the period preceding death).^15^

We selected the ITF for this study because, although most patients with GBS recover fully neurologically, recovery can be prolonged for months or years, and even those that do recover will go through an illness trajectory from symptom onset to longer term physical, psychological and social sequelae that can negatively affect their quality of life.^6^, ^16^

We used purposive sampling to recruit volunteers using the following inclusion criteria: people with a prior diagnosis of GBS, who had been discharged from hospital; able to give informed consent; able to speak and understand English; and resident in the United Kingdom. We also selected participants on the basis of age (adults aged 18 years or over), sex, marital status, location, time since diagnosis, and length of hospital stay, to ensure variation in these characteristics and a range of experiences.

We recruited eligible individuals via social media and through the Guillain‐Barré and Associated Inflammatory Neuropathies (GAIN) charity website and their social media pages. There were instructions in the advertisement for interested potential participants to contact the research team via telephone or email. On contacting the researcher(s) to express interest in taking part in a face‐to‐face or telephone interview (according to the participant's preference), study information sheets and consent forms were emailed to potential participants. Verbal or written consent was received from all participants prior to the interviews. There were no dropouts or refusals to participate once the sample was recruited.

### 2.2 Data collection

We gained ethical approval from the University of Lincoln ethics committee. We collected data using individual face‐to‐face or telephone semi‐structured interviews, based on the participants' preference and following informed consent. The interview schedule was piloted with the first two participants with no changes required to the schedule. Each interview lasted from 45 minutes to one hour and was audio recorded with participants' consent.

### 2.3 Data analysis

The audio‐recorded data were transcribed verbatim, entered into NVivo 11 and analysed using the Framework Method of thematic analysis.^17^ We used the Illness Trajectory Framework (ITF) as a lens through which to understand the participant's journey from illness onset to their current state of health.

The interviewer, a clinical nurse with expertise in qualitative methods by background, was not an expert in GBS and had no prior relationship with the participants; he sought to maintain an open mind and ensure his personal views and knowledge did not influence the views of participants. The wider academic team consisted of an academic general practitioner, two qualitative researchers, a behavioural psychologist and cognitive neuroscientist who took part in the analysis.

The analysis firstly involved all team members familiarizing themselves with the data by reading and re‐reading transcripts. Guided by the phases of the ITF, the data were then coded by the interviewer and a qualitative researcher. Codes were discussed with the research team and developed iteratively, through further interpretation and discussion with the research team who met several times to identify descriptive and then higher order or overarching themes.

The Consolidated Criteria for Reporting Qualitative Studies^18^ (see Table S1) was followed, to ensure transparency and trustworthiness in reporting our research. Further, we maintained an audit trail of the data analysis process (details of parent codes, descriptive and higher order themes in Table S2), adding to the trustworthiness of the results.^19^, ^20^, ^21^

Data saturation was reached after the analysis of data from 16 participants, meaning no new codes or themes were being generated.^22^ Therefore, we decided not to recruit more participants.

## 3 RESULTS

### 3.1 Participants

All participants were resident in the UK at the time of the interview. Most participants (10/16) opted for a telephone interview with a few (6/16) opting for a face‐to‐face interview at their own homes.

Participants' ages ranged from 30 to 79 years. There were slightly more male participants than female, most participants were white, with the majority being married or cohabitating. Most people had been diagnosed with GBS for 2 years or fewer, followed by those diagnosed for 10 years or more, and on the whole, most people spent 16 weeks or more in hospital before discharge. Details of participant characteristics are in Table 1.

**TABLE 1:** Characteristics of participants

Participant details		No of participants
Location	East Midlands	2
	North‐west and Cumbria	2
	South‐east	2
	London, Oxford and Central	3
	West Midlands	1
	Yorkshire	4
	Northern Ireland	1
	Wales	1
Age	30‐39	4
	40‐49	7
	50‐59	‐
	60‐79	5
Sex	M	9
	F	7
Ethnicity	White	15
	(Black, Asian and minority ethnic (BAME)	1
Marital status	Married/cohabitating	12
	Single/separated/divorced/widowed	4
Interview type	Face‐to‐face	6
	Telephone	10
Time since diagnosis	Up to 2 y	6
	2‐4.9 y	2
	5‐9.9 y	3
	10 y or over	5
Length of initial hospital stay	Up to 4 wk	3
	4‐9.9 wk	3
	10‐15.9 wk	4
	16 wk or over	6

### 3.2 Themes

Through the process of analysis, we identified six overarching themes, which covered the illness journey from experiencing the first symptoms to recovery, and aligned with the trajectory onset, crisis, acute and stable phases of the ITF (Table 2). Two further themes (communication; awareness, knowledge and information provision) encompassed the whole illness journey.

**TABLE 2:** Illness Trajectory Framework (ITF) phases and patient experience themes

ITF phase	Patient experience theme
Trajectory onset phase (onset of signs and symptoms and diagnosis)	*Importance of early diagnosis*
Crisis phase (potentially life‐threatening)	*Experience of inpatient care* *Active support for recovery* Health‐care factors that helped/hindered recovery (eg, appropriate investigations and treatment, adverse effects of treatment) Disease factors that helped/hindered recovery (eg, being fit/active prior to gbs, residual physical effects) Psychological factors that helped/hindered recovery (eg, being positive, residual psychological effects) Prior health, self‐care and lifestyle Social and spiritual factors that helped/hindered recovery (eg, peer support, faith, stigma of disability) Occupational factors that helped or hindered recovery (eg, unable to work, supportive employer)
Acute phase (symptoms controlled by a prescribed treatment regimen)	
Throughout	*Communication* *Awareness, knowledge and information provision*
Stable phase (symptoms controlled)	*Redefining recovery*

Themes are presented in the following order: importance of early diagnosis (trajectory onset phase); experience of inpatient care (crisis phase); active support for recovery (crisis and acute phases); communication; awareness, knowledge and information provision; and redefining recovery (stable phase). These are described in detail below and in Table S3.

#### 3.2.1 Importance of early diagnosis

Early diagnosis was felt to be important, enabling treatment to be started in a timely way and preventing potential complications:

Yes. There is no doubt, if the GP hadn't got it spot on first time, then I would have ended up in intensive care. It is only through that early diagnosis, that they were able to start treatment that much quicker… before it progressed so far into my body. (Participant 7)

Some participants felt they had waited too long for their diagnosis due to misattribution of symptoms or misdiagnosis:

First they thought it was meningitis or MS [multiple sclerosis]. (Participant 2)

#### 3.2.2 Experience of inpatient care

Participants described their experiences of inpatient care, which they felt affected their subsequent return to full health. This included how some health‐care professionals were caring and supportive and how this contrasted with others' less helpful behaviour:

In the intensive care unit, there was a wonderful nurse. She was absolutely fantastic: she would help me wash and braid my hair to stop it getting tangled. She would spend the most time with me to try and lip read me; but some of the other nurses where not as nice at all; very unpleasant. (Participant 1)

Another participant reflected on how they only appreciated certain aspects of rehabilitation with the benefit of hindsight: ‘Looking back now, the OTs were good. At the time they annoyed me because I was young and wanted to do it myself’. (Participant 2).

Negative experiences often related to staff attitudes to personal care, including hygiene, toileting and feeding, rather than medical treatment:

Then another healthcare assistant comes in and asks me what I want for breakfast, and then chucks the toast at you. I can't feed myself. I didn't get fed till one of my family members came in and one of them fed me. The nurses were too busy, or they didn't understand. (Participant 10)

Delays in treatment led to negative experiences and a perception that this may have also hampered recovery:

I think if I had that treatment earlier, it would have been better. (Participant 5)

#### 3.2.3 Active support for recovery

Participants described health care–related; disease‐related; psychological; prior health, self‐care and lifestyle; and social and occupational factors that helped or hindered recovery.

##### Health care–related factors

Health care–related factors that supported or hindered recovery included the following: early investigations; specific therapies; occupational and speech therapy; physiotherapy and other forms of exercise; and aids (eg, in writing).

Early investigations were believed to help specialists diagnose and commence treatments without delay, while specific therapies such as immunoglobulins, plasma exchange and analgesia were perceived as aiding recovery:

Once I got the immunoglobulin, it was quite amazing, it was almost instant after the three days, and I could lift my foot again and get out of the car. (Participant 4)

Participants reported that physiotherapy, occupational and speech therapy were helpful during hospitalization and after discharge from hospital. Exercise, such as walking or swimming, was also beneficial. Aids or equipment to help participants write, communicate or perform some other activity were also mentioned alongside other practical aids that were considered helpful in supporting daily activities:

They got me a commode because I couldn't get upstairs to the toilet; but once I was a bit stronger, they got an extra handrail, so I could get up the stairs. (Participant 2)

The health care–related factors that hindered recovery were adverse reactions to treatment, and lack of structured long‐term follow‐up care.

A participant recalled experiencing adverse reactions to treatment, which might have hindered their recovery:

I can't recall if it was when I was having my treatment or straight after, but I started getting an allergic reaction on my hands. (Participant 15)

Longer term follow‐up care was felt to be needed, but lacking:

In regard to help and recovery from the medical profession, I would say very poor. There is nothing. No hotline. No helpline. There is GB charities out there that will help you; but NHS, there is not. There must be other rare conditions out there that people who have them, get so frustrated that they just want to talk to somebody. There is no health professional out there. (Participant 12)

Some felt they were discharged too early and that further physiotherapy was beneficial when provided. When physiotherapy was stopped, some felt abandoned:

I did have community physio. That was when I suggested to the physio to see them once a month; and eventually he did agree to that. It was kind of like patient led; it wasn't the system doing it for me. Then I was discharged from the community physio. That was it. I have been left on the scrapheap. I felt like the system had let me down quite badly. (Participant 6)

Another participant reported the need for more structured follow‐up care from their GP:

I do feel that there could be somebody who would follow up on where I was; rather than me feeling I had to go to the GP when I was getting to stages where I was feeling depressed, or fatigue got worse, or not sure what was happening with my pregabalin dosages…. I feel like I was just left to sort myself out. (Participant 5)

##### Disease‐related factors

Disease‐related factors that mostly hindered recovery were identified by participants including comorbidities (‘How do you treat GBS and this…at the same time without complications on each one’ [Participant 10]); residual or late physical problems (‘I haven't made a full recovery. I still have peripheral neuropathy in my legs…I suffer with chronic fatigue as well’ [Participant 1]); and sequelae or complications of GBS that affected other body organs (‘They found that I also had all sorts of other complications because of the GBS It had attacked my parathyroid, my heart, and kidneys’ [Participant 3]).

##### Psychological factors

A number of psychological factors that may support or hinder recovery from GBS or a related condition were identified. The supportive factors included being positive and being discharged from hospital to a home environment.

Participants felt being negative could worsen the situation, whereas maintaining a positive attitude and feeling lucky to be alive could be helpful:

It is all about attitude: if your glass is always half empty, you are going to suffer terribly. The negativity will bring them down, terribly. You have to have a positive mental attitude. (Participant 3)

Being admitted to hospital for treatment of GBS was important, but being in hospital for long periods was less helpful for some who talked about how they desperately wanted to go home, and a participant stated that, ‘my first few weeks of coming home, I progressed really quickly; rather than be in a hospital environment’ (Participant 13).

Psychological problems, such as anxiety, depression or sleep difficulties, at the initial or later stages of the illness, were perceived to impede recovery. For example, a participant stated that, ‘I don't want to live if I am going to be a burden to my family; if I am going to be paralysed’ (Participant 10), and another said, ‘I still get night terrors, my partner just has to hold me; …. and I am screaming in my sleep’ (Participant 1).

Participants talked about counselling in terms of support with psychological problems. Unfortunately, participants did not always receive the counselling that they needed: ‘Having somebody to talk about my hallucinations and night terrors, I never had that sort of help’ (Participant 1). Where counselling was offered, some people waited too long for an appointment, meaning people were left feeling helpless: ‘I was referred for some counselling. Once I went to my GP it was 7 months waiting list to see anybody’ (Participant 6).

The lack of an opportunity for counselling led some people to seek their GP's support:

It is something that I am seeing my GP about this following month, because there are some days when I get really frustrated. I need to be able to deal with the fact that this could be my life now; and that I may never have my old life back. (Participant 11)

#### 3.2.4 Prior health, self‐care and lifestyle

Being young and fit, maintaining independence and keeping active, being proactive about doing things and adjusting lifestyles were thought to help with recovery. For example, a participant suggested that,

The other thing that probably helped with the recovery … I was really fit. (Participant 7)

Similarly, participants tried to help their recovery by maintaining independence and keeping active, and a participant felt being proactive about doing things helped a lot:

I try to walk a lot. I feel, in some ways, I've had to help myself. I have been pro‐active about being better. (Participant 16)

Adjusting lifestyles supported a sense of recovery. A participant, who was housebound and mostly alone, described how they managed to do everything they needed to:

Well because I was more or less home on my own, I just managed to potter about and slowly do everything I needed to do in the house, ok. I learned how to use the internet and do internet shopping for everything. (Participant 9)

#### 3.2.5 Social and spiritual factors

Social and spiritual factors supporting recovery included religion; social media; sharing experiences with others; and support from family and friends.

Religion and faith‐based practices helped some participants to cope: ‘A lot of it was my faith…as a Buddhist, we chant; and I chanted in my head’ (Participant 1).

Similarly, social media, for example Facebook, helped to link participants up with other people diagnosed with GBS. A participant suggested that, ‘If it wasn't for social media I wouldn't have met A, and gone to see his production’ (Participant 2), and another participant found, ‘the support group on Facebook and reading other people's stories helpful’ (Participant 11).

Participants also found sharing experiences with others useful for themselves and other GBS patients:

I now volunteer through the GAIN charity. I go into hospitals and I visit people. There is a young mum recovering from GBS, and she wanted someone to talk it over with; ……. I have made so much progress and so can she. (Participant 6)

Support from family and friends by having someone to talk to and provide encouragement in times of loneliness and worry was also found to be helpful. A participant stated that, ‘It certainly helps having good family support’ (Participant 7). Another participant suggested that a desire to socialize and live a normal life again with family and friends also helped:

I've had a lot of support from my family – my children. I have lots of friends. I wanted to be able to socialise again. I wanted to be able to go home and cook and garden and lead a normal life again. I just feel my recovery has been helped by family and friends. (Participant 16)

The social factors cited as hindering recovery included impact on family members, marriage and other relationships; limited social care support; lack of social life; and stigma.

The illness sometimes had a negative impact on families and this, in turn, affected recovery. One family was terrified of losing their affected member (the participant) after being told of the possible outcomes of GBS: ‘They also told the family that most people recover, but sometimes people don't. So, they were all terrified that I was going to die’ (Participant 9).

Another participant described how his illness and admission to hospital impacted negatively on his wife: ‘I think it is starting to take its toll on my wife. She is starting to get a bit run down and unwell herself’. (Participant 13). Sometimes the illness led to marital or relationship breakdown. This was sometimes initiated by the participant's partner, as one participant said,

My illness is why I am getting divorced; my ex‐husband couldn't handle the fact that I wasn't the old me. (Participant 1)

Similarly, a participant decided to end a relationship after getting GBS, because he had changed, and his partner had struggled to cope. Another participant acknowledged that their ability to maintain relationships was not as good as before:

Marital relationships are not; no longer doable at the moment. There is slight mood swings, but I think that is probably medication. (Participant 7)

Social care support was important for some affected. One participant talked about struggling with carer responsibilities and suggested that there was limited support for the children of disabled parents:

Just basic things I would like to be able to do with him that I just can't do. Interestingly, I phoned up children's services to see if there was any help that they could give; and there is no group for children of disabled parents. (Participant 1)

Some participants had to wait too long for equipment from social services, but were readily provided a grant for equipment by a charity:

The NHS mentioned an electric wheelchair, but they said it can take up to 12 months to get one. GAIN said they might be able to help. They sent us an application form, and they paid for my electric wheelchair. That was fantastic. (Participant 13)

Furthermore, although resources for people with disability could help people recovering from GBS, some social care resources were means tested, meaning some with GBS did not get the support they needed. A participant said:

I asked them to adapt the bathroom…and they said that it was all means tested and because I had savings,…Basically what you want me to do now is the savings that me and my wife had, is use all of that, so we have no savings left, and then what do we do? (Participant 12)

Some participants appeared unhappy that they could not socialize after getting GBS, which, in turn, did not help with recovery. One participant said, ‘socially my life has come to a complete stand‐still’ (Participant 4). Another believed their life had been turned upside‐down:

In terms of how my life was pre‐ GBS, it has just been turned upside‐down. Before I had GBS, we were moving into a new build house. We hadn't been on holidays. All that is gone. (Participant 12)

Stigma was another factor that hindered recovery, preventing some people from seeking help:

I think the one big problem I did have, I suppose, was my own pride. I didn't like to be seen as disabled. I didn't like that label put on me, but unfortunately that is how I was labelled, and to a certain extent, possibly seen to be even now. (Participant 4)

Similarly, a participant avoided crowds, because he did not want to be seen in a wheelchair:

Yeah, I don't tend to go out much. I don't like crowds. I get embarrassed a bit when I see people I haven't seen for years. They see me in a wheelchair. It is a kind of a stigma thing. (Participant 12)

### 3.3 Occupational factors

Occupational factors such as supportive employers and/or work colleagues and in‐work benefits promoted recovery. Employers and work colleagues could help people returning to work after their illness by being supportive and showing understanding. For example, a participant found a flexible work schedule helpful: ‘My Company were extremely supportive. They gave me little bits of work back at a time. I wasn't stressed or loaded on’ (Participant 11). Some employers allowed people to change their work pattern from full time to part time.

While benefits may not have been enough for some, they were a support for some participants who had returned to work and were struggling with transport as they were unable to drive: ‘There was no way I could drive anyway, but I managed to get a grant for a taxi to work every day and back again’ (Participant 5).

Occupational factors that were perceived as hampering recovery included being forced to stop working due to the illness and the financial burden of not working. Following the onset of GBS, some people could not work even on a part‐time basis and were forced to retire: ‘I never got back to work. I wasn't able to go to work’ (Participant 4). Others had to give up self‐employment: ‘I haven't been to work ever since it happened. I was working for myself’ (Participant 3). As a result of not being able to work as before, some people experienced some degree of financial burden: ‘Honestly, going from a £50K a year job down to benefits…’ (Participant 1).

#### 3.3.1 Communication

Effective communication was considered to be important throughout the illness trajectory, both during the acute phase and recovery. However, many participants talked about unhelpful communication experiences, due to lack of information about their condition and because they were not told what to expect. For example, one interviewee felt that little was communicated to her until she had a tracheostomy in place and could not speak, leaving her confused about her diagnosis:

I wasn't told anything. I wasn't told what I had or what was going to happen. I remember Dr S, he said – “Oh you are really lucky, you have GBS!” I thought I've never heard of GBS. That is when I found out what I had, but I still had absolutely no idea what that was. (Participant 1)

Poor communication was worse when the condition affected speech:

I think communication, when you can't speak, is really difficult. They had to lip read me. Some people would bother, and some wouldn't. You couldn't tell people when you were in pain or wanted to go to the toilet. (Participant 1)

Others felt they were not listened to or taken seriously:

Yes, because they weren't listening to me saying I knew there was something wrong with me. They were just saying take Diazepam, rest. How ill I felt, I knew it was more than that. (Participant 11)

#### 3.3.2 Awareness, knowledge and information provision

Participants identified elements of awareness, knowledge and information provision as lacking throughout the illness journey. According to one participant, social media was their main source of information:

It is the awareness that needs to happen. I see about it every day because I follow it all on Facebook, but if there weren't any social media, even less people would know about it. (Participant 2)

Another participant agreed that there was limited effort to provide useful information. Although GBS was rare, it was felt that better information should be provided for patients:

The only thing that didn't help was there isn't a lot of knowledge out there. I think we need something out there to support people and their families. It is different when you watch someone with cancer or something because we are so well informed on it. (Participant 7)

Participants also appreciated the role of charities, such as GAIN, in providing information and support for people with GBS and related conditions, and suggested that people should be informed about GAIN and other social media websites as a source of information:

I do think it is important that they are advised about the charity, because the charity plays a great role in helping patients who are frightened; who have never experienced anything like this before. (Participant 4)

#### 3.3.3 Redefining recovery

Participants redefined recovery and expressed achieving ‘a new normal’, which meant adjusting or changing their daily activities. One participant found it helpful to make mental and physical changes to get on with life in the short and long term. While doing so, there was a recognition that their future was going to be different:

You have to adjust mentally and physically because there are things that I can't do that I have to get other people to help me with. I liken it to the soldiers who have come back from Afghanistan who have had their legs blown off…. They still have a life, but it is a different life. (Participant 3)

Recovery meant different things to different participants. One participant said, ‘Well, I'm walking. That to me is recovery’ (Participation 10). Another stated: ‘It is getting back to as near normal as possible; apart from the physical side. Sunday, my wife and I went down to the village – it was nice; because I had got my electric wheelchair, I was able to hold her hand while we walked down to the village. That to me is getting back to normal’ (Participant 13).

## 4 DISCUSSION

This study explored the experiences of people with GBS and identified the importance of early diagnosis, better experiences of inpatient care, active support for recovery, good communication, and improved information provision by health‐care staff to support those affected to recover from their illness. This led affected individuals to redefine what recovery meant for them.

Although the Illness Trajectory Framework (ITF) informed the data collection and analysis, as a result of the themes arising from the analysis, we used Response Shift Theory to complement the ITF in helping to explain key findings. Response shift theory^23^ helps understand how people experiencing a rare and serious condition like GBS progress from diagnosis to recovery as a positive adaptive process through five key concepts: catalyst, antecedents, mechanisms, response shift, and perceived quality of life.^23^ The catalyst is the illness or change in an individual's condition resulting from treatment. Antecedents refer to attributes such as sociodemographic characteristics, personality, expectations and spiritual identity. Mechanisms refer to the person's response to their illness through coping strategies, social support, goal reordering, reframing expectations and spiritual strategies. The presence of a catalyst (the illness) can trigger a person's antecedents and mechanisms into play, leading to a response shift (that involves a change in internal standards, values and conceptualizations) which lead to a change in perceived quality of life.^23^ The theory helps to explain many of the findings discussed below.

Participants felt that early diagnosis and treatment with intravenous immunoglobulin (IVIg) or Plasma Exchange (PEX) helped to speed up their recovery and prevented potential medical complications. This is supported by evidence from a systematic review of randomized controlled trials showing that early administration of IVIg or PEX significantly hastened recovery from GBS,^24^ whereas the evidence for corticosteroids and other agents is limited and of low quality.^25^ There is little evidence for long‐term benefits from immunotherapy.^26^

Although some participants experienced positive care practices from health‐care professionals, others had negative experiences which they felt adversely affected their progress towards recovery. Safe and effective inpatient care was reported to improve patients' expectations and strengthen coping strategies or change understanding of their health status. This helped to promote recovery or for individuals to regain normal function and improved perceived quality of life, as expressed in the response shift theory.^23^ Although the importance of early diagnosis and effective inpatient care are not new concepts in many disease situations, these findings are novel in terms of the experiences or perceptions of people with GBS and our understanding of the needs and expectations of people with this or related conditions.

Health‐care factors that were perceived to support recovery correspond with evidence that rehabilitation and exercise help GBS patients to regain independence by strengthening weakened muscles and enabling them to relearn daily activities.^12^, ^27^ Adverse effects of treatment and lack of longer term follow up care, particularly for patients with persistent residual problems such as pain, chronic fatigue and disability, were perceived to hinder recovery, which points to the importance of providing structured follow‐up care for GBS patients after hospital discharge and conducting well‐designed evaluations of such provision.^28^

Previous long‐term studies have found that residual problems adversely affected quality of life in adults recovering from GBS.^6^, ^7^, ^9^, ^10^ For example, Bersano et al from interviews with 70 patients 3‐5 years from onset of GBS found that most participants (64%) made a complete functional recovery, while others (27%) were independent with only minor limitations in daily activities, and a few (9%) needed help during the day; importantly, over a quarter ( 27%) had substantial changes in their job, hobbies or social activities.^7^ Neuropathic pain was also found to affect over a third of people with previous GBS29 and fatigue is another common symptom.^2^

Psychological disorders associated with GBS such as anxiety, depression, post‐traumatic stress disorder, insomnia and other sleep disturbances^29^, ^30^ can hinder recovery. A positive attitude to recovery was seen as helpful and was consistent with GBS being perceived, at least at the initial stages of the illness, to be a temporary condition with a predictable outcome of recovery.^31^, ^32^ As response shift theory suggests,^23^ a positive attitude in this situation is a helpful coping strategy that encourages an individual's resolve or determination to get well. Receiving counselling and being discharged from an inpatient care setting to a home environment were also perceived by participants as supporting the recovery experience, as these helped to improve patients' expectations of recovery, with social support from family and friends also increasing coping, well‐being and quality of life. In contrast, anxiety, depression or sleep difficulties, at the initial or later stages of the illness were perceived as hindering recovery.

Previous studies have shown that older age, severe disability at admission and nadir, ventilator dependence and type of nerve dysfunction affect long‐term prognosis.^27^ Participants in this study also perceived that being younger helped recovery but they also felt that prior health status and fitness, together with self‐care and lifestyle factors including a focus on maintaining independence and keeping active, being proactive about maintaining function and adjusting lifestyle to condition improved the experience of recovery. These findings add to our knowledge and understanding of the needs of GBS patients to regain independence.

Social factors that supported or hindered the recovery experience was also expressed by participants in this study. Attending faith‐based meetings helped recovery and coping with the illness. This related to the concept of spiritual practices or behaviours in response shift theory^23^ which suggests that spiritual practices or beliefs could serve as coping strategies for illness recovery. Social media, sharing experience of GBS with others or peer support, support from family and friends, and support from social care, etc, that also facilitated recovery, are described collectively in response shift theory as social support mechanisms for recovery.^23^ Participants also mentioned factors they perceived to hinder their experience of recovery, for example, the stigma of being seen in a wheelchair and a perceived negative impact of having GBS on marital or other relationships. This suggests mechanisms such as coping, social support or ability to reframe expectations on the part of the individuals involved^23^ are failing or being overwhelmed by the illness. Stigma of being seen in a wheelchair as perceived by participants in our study is consistent with previous research, which found a stigma related behaviour whereby participants dealt with potential loss of self by trying to conceal their impairments, or by not discussing them with others at their place of work.^13^ With the exception of stigma, the perceived social factors that support or hinder the recovery experience are new and represent an addition to knowledge in terms of the experiences and needs of people recovering from GBS.

The opportunity to return to a supportive working environment helped patients to cope and reorder their goals,^23^ whereas early retirement due to the illness was perceived to further hinder recovery. In a previous study, 62% of patients were able to return to their former employment post‐GBS, with the rest needing alterations at work to cope with the physical demands of their roles.^33^ In other studies, fewer than half of GBS patients returned to work within two years,^9^ with some only managing reduced hours or part‐time work^6^, ^33^ due to loss of muscle power, muscle pain, disturbed sensation and fatigue.^7^, ^33^, ^34^ Unemployment or retirement resulted in financial burdens not alleviated by benefits.^13^

Effective communication is a known factor in patient satisfaction, complaints and integral to high‐quality health care.^35^ Participants in our study expressed concerns about poor communication and information provision. Increasing information provision to the public and professionals about GBS would help to engender understanding of the physical and other residual problems affecting people recovering from GBS^13^ and pave a way for appropriate support systems to be accessed by people who need them.

Colleagues at work made an important contribution to people with the condition feeling ‘normal’ again.^13^

### 4.1 Strengths and limitations

This is the first qualitative study exploring the experiences of people in the UK with GBS from symptom onset to recovery. A recent qualitative study in this area identified a gap in the evidence in relation to the long‐term physical and psychosocial experiences of individuals with GBS^14^ and our findings bridge this gap. The academic nurse who conducted the interviews had no prior clinical contact with participants, and the team included a range of clinical and academic expertise. The Consolidated Criteria for Reporting Qualitative Studies^18^ was followed, to ensure transparency and trustworthiness in reporting our research.

The illness trajectory of 16 people with GBS may not be a true representation of people with GBS in the UK as we did not assess or recruit on the basis of disease severity, but we did reach data saturation after interviewing and analysing data for 16 participants. We used the ITF to help explain the illness journey of GBS patients in our study, from symptom onset through to recovery. Although we acknowledge that the ITF was designed for a chronic illness trajectory, rather than an acute condition with neurological recovery in most GBS patients, we heard from participants in this study that they experienced long‐term physical, psychological and social symptoms. Some participants included in the study had severe residual disabilities requiring aids such as a wheelchair and this may have represented a subgroup of patients who also had more difficult psychological and social experiences of recovery. Nevertheless, we wanted to explore the range of experiences of patients with GBS rather than the experiences of a representative sample and our approach reflected this approach.

It was not within the scope of this study to assess associations between the findings and participant characteristics, such as age, sex and ethnicity, and we did not explore the functioning and severity of disability of participants. We also did not explore the views of health‐care professionals and family members or carers of people with GBS in this study. These will need to be considered in further research. We were unable to confirm the diagnosis of GBS via medical records and relied on the use of screening questions on recruitment to interviews to recruit participants with a self‐declared diagnosis of GBS. Certain participant characteristics such as time since diagnosis and length of hospital stay may have been subject to recall bias.

### 4.2 Implications for practice, policy and future research

Early detection and treatment of GBS to facilitate recovery and prevent complications is important to patients but structured follow‐up care and rehabilitation, appropriate to the medical and social care needs of individual patients following discharge from hospital, should also be planned and implemented more widely for those with GBS.^36^ Rehabilitation has been shown to be effective,^37^ but outpatient provision may not always be available to those who could benefit.^38^

More needs to be done to raise awareness among health‐care staff about rare but serious conditions such as GBS and provide appropriate training where this is needed. Greater knowledge among health‐care staff would benefit GBS patients, their families and informal carers and signposting to further information and support would also be valuable. Charities such as GAIN are currently offering a range of support and advice services, which are valued by patients with GBS,^36^ but more could also be done through centres of expertise to provide information and support to both patients, families and staff.

Although GBS is a monophasic disease and patients often achieve a neurological recovery, residual symptoms such as neuropathic pain and fatigue, with causes secondary to or complicating the primary neurological pathology, may occur. Fatigue may respond to exercise^39^ and neuropathic pain to drugs^36^ but further research to explore treatments and strategies to alleviate these residual symptoms is important for improving experience and quality of life (QOL) of patients with GBS.

Future research should also explore the perspectives of health‐care professionals, and family members or carers of people with GBS. While the current interview study provides an in‐depth insight into the experiences of the study population, further larger scale quantitative studies are recommended to provide further evidence about current and future health‐care provision and support for individuals with GBS and related conditions. As the emphasis on self‐care and peer or other support services increases, it would be helpful to understand how these could be optimized.

## 5 CONCLUSION

The key themes identified within this paper have provided a framework to enable us to begin to understand the perceived factors that helped or hindered the participant's journey from symptom onset through to recovery. In exploring the experiences of individuals with GBS, we can develop a better understanding of the care and support that they require.

## CONFLICT OF INTEREST

The authors have no competing interests to declare.

## AUTHORS' CONTRIBUTIONS

ANS had the original idea for the study. The study was designed by ANS and JNA, supported by DL, FC, JJ and TLH. Fieldwork and analysis were conducted by JNA supported by ANS, DL, FC and JJ. JNA wrote the first draft of the paper, and all the authors edited and approved the paper.

## ETHICS APPROVAL AND CONSENT TO PARTICIPATE

The study was approved by the Lincoln University Ethics Committee. All interviewees gave informed consent to participate. The study was performed in accordance with the Declaration of Helsinki.

## Supporting information

Table S1

Table S2

Table S3

## Data Availability

The data analysed during this study are available from the corresponding author on reasonable request.
