# Supplementary material for: Exploring the experiences of having Guillain‐Barré Syndrome: A qualitative interview study
Source: Health Expect. 2020 Aug 3;23(5):1338–49. doi: 10.1111/hex.13116 (PMC7696117; doi:10.1111/hex.13116)
Supplement: Supplementary file 1 — Table S1 [file HEX-23-1338-s001.docx]

**Table S1: Consolidated criteria for Reporting Qualitative research (COREQ) Checklist**

| **No. Item** | **Guide questions/description** | **Reported on Page** |
| --- | --- | --- |
| **Domain 1: Research team and reﬂexivity** |  |  |
| *Personal Characteristics* |  |  |
| 1. Interviewer/facilitator | Which author/s conducted the interview or focus group? | JNA, page 4 |
| 2. Credentials | What were the researcher’s credentials? E.g. PhD, MD | MPH MPhil, PhD |
| 3. Occupation | What was their occupation at the time of the study? | Research assistant |
| 4. Gender | Was the researcher male or female? | Male |
| 5. Experience and training | What experience or training did the researcher have? | JNA had training and experience of research interviews to collect and analyse qualitative data (page 4). |
| *Relationship with participants* |  |  |
| 6. Relationship established | Was a relationship established prior to study commencement? | Yes, the researcher introduced himself and interacted briefly with participants as part of the preparation for the interviews. |
| 7. Participant knowledge of the interviewer | What did the participants know about the researcher? e.g. personal goals, reasons for doing the research | As a researcher with interest in healthcare improvement. |
| 8. Interviewer characteristics | What characteristics were reported about the interviewer/facilitator? e.g. Bias, assumptions, reasons and interests in the research topic | The interviewer, a clinical nurse with expertise in qualitative methods by background, was not an expert in GBS, and had no prior relationship with the participants; sought to maintain an open mind and ensure his personal views and knowledge did not influence the views of participants. The wider academic team consisted of an academic general practitioner, two qualitative researchers, a behavioural psychologist and cognitive neuroscientist who took part in the analysis (Page 4).  Pages 19-20: The academic nurse who conducted the interviews had no prior clinical contact with participants, and the team included a range of clinical and academic expertise. |
| **Domain 2: study design** |  |  |
| *Theoretical framework* |  |  |
| 9. Methodological orientation and Theory | What methodological orientation was stated to underpin the study? e.g. grounded theory, discourse analysis, ethnography, phenomenology, content analysis | We used the Framework Method of thematic analysis (Richie and Spencer, 1994) and ‘the Illness Trajectory Framework (ITF) to follow the participant’s journey from illness onset to their current state of health’ as stated on page 4. |
| *Participant selection* |  |  |
| 10. Sampling | How were participants selected? e.g. purposive, convenience, consecutive, snowball | Purposive sampling as described on page 3. |
| 11. Method of approach | How were participants approached? e.g. face-to-face, telephone, mail, email | Approached via advert circulated via GAIN charity website and social media followed by telephone screening and face-to-face or telephone individual interviews as stated on page 3-4. |
| 12. Sample size | How many participants were in the study? | 16 participants as stated on page 4. |
| 13. Non-participation | How many people refused to participate or dropped out? Reasons? | There were no dropouts or refusals to participate once the sample was recruited, page 4. |
| *Setting* |  |  |
| 14. Setting of data collection | Where was the data collected? e.g. home, clinic, workplace | In service users’ homes or by telephone, depending on service users’ preference (page 4). |
| 15. Presence of non-participants | Was anyone else present besides the participants and researchers? | No. |
| 16. Description of sample | What are the important characteristics of the sample? e.g. demographic data, date | Please see Participants Characteristics in Table 1. |
| *Data collection* |  |  |
| 17. Interview guide | Were questions, prompts, guides provided by the authors? Was it pilot tested? | Yes, the interview schedule was piloted with the first two participants with no changes required to the schedule (page 4). |
| 18. Repeat interviews | Were repeat interviews carried out? If yes, how many? | No |
| 19. Audio/visual recording | Did the researcher use audio or visual recording to collect the data? | Yes, as stated on page 4. |
| 20. Field notes | Were ﬁeld notes made during and/or after the interview or focus group? | Yes |
| 21. Duration | What was the duration of the interviews or focus group? | 45 - 60 minutes per interview, page 4. |
| 22. Data saturation | Was data saturation discussed? | Yes, as stated on page 4 |
| 23. Transcripts returned | Were transcripts returned to participants for comment and/or correction? | No |
| **Domain 3: analysis and ﬁndings** |  |  |
| *Data analysis* |  |  |
| 24. Number of data coders | How many data coders coded the data? | 2 people (JNA, DL) coded the data (the same transcripts) independently. |
| 25. Description of the coding tree | Did authors provide a description of the coding tree? | Yes, as in Supplementary Table S2. |
| 26. Derivation of themes | Were themes identiﬁed in advance or derived from the data? | Themes were identified from the data but the identification process was guided by the theoretical framework informed the study as described on page 4. |
| 27. Software | What software, if applicable, was used to manage the data? | NVivo software as stated on page 4. |
| 28. Participant checking | Did participants provide feedback on the ﬁndings? | No |
| *Reporting* |  |  |
| 29. Quotations presented | Were participant quotations presented to illustrate the themes/ﬁndings? Was each quotation identiﬁed? e.g. participant number | Yes, in Results on pages 5-15. |
| 30. Data and ﬁndings consistent | Was there consistency between the data presented and the ﬁndings? | Yes, in Results on pages 5-15. |
| 31. Clarity of major themes | Were major themes clearly presented in the ﬁndings? | Yes, in Results on pages 5-15. |
| 32. Clarity of minor themes | Is there a description of diverse cases or discussion of minor themes? | Yes, in Results on pages 5-15. |

Developed from: Tong A, Sainsbury P, Craig J. Consolidated criteria for reporting qualitative research (COREQ): a 32-item checklist for interviews and focus groups. International Journal for Quality in Health Care. 2007; 19, 349 – 357.
