# Supplementary material for: Exploring the experiences of having Guillain‐Barré Syndrome: A qualitative interview study
Source: Health Expect. 2020 Aug 3;23(5):1338–49. doi: 10.1111/hex.13116 (PMC7696117; doi:10.1111/hex.13116)
Supplement: Supplementary file 2 — Table S2 [file HEX-23-1338-s002.docx]

**Audit trail of the data analysis process**

In total, 16 semi-structured interviews were conducted. These lasted between 45 to 60 minutes, were audio-recorded with consent of participants and transcribed. With the help of an NVivo software, the data were analysed using a thematic framework approach. The research team familiarised themselves with the data by carefully reading the transcripts. Guided by the Illness Trajectory Framework (ITF), initial or parent coding of the data was performed. Further coding and discussion with the research team led to the development of descriptive and higher order themes and related sub-themes. Details of the initial codes, descriptive and higher order themes are presented in Supplementary Table S2 below:

**Table S2 Audit trail of data analysis process (parent codes, descriptive and higher order themes)**

| **Higher order themes** | **Descriptive codes/themes** | **Parent codes** |
| --- | --- | --- |
| Importance of early diagnosis | The early detection by GP and hospital helped with recovery | Went to see GP who suspected GBS and referred to hospital  Early detection with early treatment would be better |
|  | Misattributing symptoms in primary care/ Misdiagnosis | GP said to go on holiday  GP said numbness around waist was probably due to stress  GP recommended rest or holiday for tingling in toes, finger tips and tongue  GP thought it was motor neurone disease  Before the admission and lumbar puncture, people said it was backache |
|  | Misattributing symptoms in hospital  / Misdiagnosis | Brain tumour and broken neck were the initial diagnosis in Spain  Not sure about the cause but suspected Lyme disease  Health staff didn't understand GBS symptoms and thought patient was exaggerating  With second episode, A&E thought it was stroke or TIA, so was discharged home  Went to A&E suspected it was cannabis, and discharged home for it to clear  Was discharged from hospital and referred to Psychiatrist and then referred to Neurologist who then diagnosed GBS  Was discharged home from A&E thinking illness was a stroke or TIA  They thought it was meningitis when the illness first started  Numbness was dismissed as being melodramatic  Suggested to doctor that it was GBS but doctor dismissed it  Still feel ill after visiting A&E, GP prescribed hydrating tablets for diarrhoea and advised plenty of rest and water  Some can have it and if not severe, it can get swept under the carpet  Part of the reason for some being in hospital for long could be poor diagnosis in the early days  On first admission to hospital, consultant couldn't help and didn't decided to discharge because MRI scan was clear  GP had queried AIDP which hospital staff didn't seems to notice  GP suspected AIDP but hospital staff thought it was a breathing problem  Hospital staff suspected a breathing problem instead of GBS  GBS was diagnosed late because GPs and A&E doctors had seen and thought it was stress or anxiety  Everybody thought the swollen legs was lymphoedema but it was not  After discharge from hospital, went to see Psychiatrist but can't remember who did the referral  Became ill in October 2001 but GBS not diagnosed until January 2002  Initially thought the illness was due to old spine problems, so doctors did test around this  Eventually got diagnosed with GBS after admission to hospital through 999 call  Felt worse and GP referred to A&E and discharged because couldn't find anything after various test  Following visit to GP and A&E, condition worsened and returned to hospital by ambulance  Got admitted to respiratory ward after a series of tests  Got sent back home again after various tests at A&E  GP kept saying to take painkillers because they thought it was backache  Had asked consultant why it took longer to diagnose GBS as compared to first time  Had the illness the second time and they were quite slow in diagnosing it  There was no confirmed diagnosis while on holiday, only upon return to UK and lumbar puncture was done  Was referred by GP to hospital but GBS was not detected until seen by neurologist who did lumbar puncture  About 6 months prior to GBS, had hurt rib and coughed a lot |
|  | Delayed diagnosis | It was after nerve conducting study on legs that they realised it was GBS and not spine problem  Upon return from holiday, symptoms worsened and GP referred to hospital  The delay in diagnosing GBS as compared to the first time is a reflection of the size and mode of operation of hospitals these days |
| Experience of inpatient care | Good care by some NHS staff | Appropriate treatment provided  Hospital staff, nurses, doctors, physios and OTs helped with recovery  Some health professionals like physio were very knowledgeable about GBS but others were not  Wide variation in nursing care  Once the GBS was diagnosed the treatment and care was great  Went to see doctors on how to prevent recurrence, but was rediagnosed with CIDP in 2015  Pressure to release hospital bed  Used to have a walk outside with some doctors |
|  | Delay in starting treatment till consultant confirmed diagnosis | Although GBS was detected early, treatment delayed until consultant came to confirm  Although they knew it was GBS, the treatment didn't start until the tests confirmed |
|  | Poor nursing care | It was difficult to find a decent nurse to look after you  It was not a good experience being in hospital and just wanted to go home  It was not helpful that nurses expected a paralysed person to do things  The neglect was traumatising then and now  There is neglect of people with paralysis by hospital care staff  Was ignored by nurses during hallucinations and delusions  Was in pain, but they thought I was a drama queen and 'pain in the arse' |
|  | Negative experience with physiotherapy and lumbar puncture | Negative experience with lumbar puncture  The physio involving walking on crutches was very agonising due to the osteoporosis linked to the GBS, but the doctors and physios wouldn't understand |
| Active support for recovery | **Health care factors** | |
|  | Appropriate investigations in GBS | Doctor did assessment - a nerve conductor test which showed signals were getting through  Someone heard me speak and went to hospital because they recognised the symptoms  They found out after the nerve conduction test that I had no nerve left in my legs  They did a lumbar puncture that confirmed their suspicion of GBS  Neurologist diagnosed GBS after lumbar puncture and nerve conduction tests  At the Neurologist referral, nerve conduction test and lumbar puncture led to diagnosis of GBS  Had lots of tests including lumbar puncture to help detect GBS  Had tracheostomy on critical care unit and was under sedation for up to 5 days  Correct diagnosis by lumbar puncture  Lumbar puncture test confirmed it was GBS  Was flown from Spain to UK on advice of UK doctor who suspected GBS  Early diagnosis by English doctor in Denmark and treatment with plasma exchange helped with recovery  Was taken to hospital and one consultant diagnosed GBS after some tests  Was flown back from the Spain to the UK and straight to intensive care  A neurologist suspected GBS confirmed with a lumbar puncture |
|  | Physiotherapy and others (OT, SALT) | Advice for others - engage with physio as much as possible and ask somebody  All support including OT and physio should be offered for long term  Did own house assessment because disability was one of the things from past working experience at MOJ  Discharged from hospital with physiotherapy and gym 3 times a week, then swimming twice a week  Discharged home with only a wheelchair and commode, but was asked to pay for bathroom to be modified  Physio led bed exercises  Had a lot of physio exercises after discharge from hospital and movement is now quite good  Had hydrotherapy as part of physiotherapy  Had physio at rehab centre after discharge from hospital  Had physiotherapy after leaving hospital which was extremely tiring  Had rehabilitation in hospital  Had some follow up from physio after asking for it, which was patient led rather than the system doing it  Has been lifting weights by himself and with physio at home  Initially saw physios at home twice a week dropping to once a week as condition improved, then stopped seeing them  It has been a very long slow recovery pushed through physiotherapy, otherwise would still be in a wheelchair  It would have taken longer to recover without physio  Lots of exercises every week for 6 months with and without physiotherapist helped with recovery  Moved to rehab after hospital and physio wasn't as much as expected  OT and speech therapist were very good in reassuring and supporting with ADL  Physio really helps but need to push for good physio  Physio started in hospital and continued after discharge 4 days a week  Physios followed up at home and did really well  Physiotherapy and keeping active is really helpful  Physio (passive movement) helped a lot with the pain  Didn't appreciate it at the time but on reflection, the OTs were good |
|  | Treatment with Immunoglobulins, plasma exchange and analgesics | Also had steroid treatment in addition to IVIG but can't remember which steroid  An infusion of a drug for the osteoporosis also resulted in arthritis and replacement of both hip  Had a 2 five day courses of Intravenous Immunoglobulin (IVIG) and some steroids  Had Immunoglobulins for treatment of GBS  Had immunoglobulins on respiratory but continued to deteriorating and ended up in critical care  Had immunoglobulins the second time of GBS which might has helped to resolved double vision symptom quicker than the first episode of GBS  Had IVG drip as day patient first time GBS attacked, which helped  Immunoglobulins had to be brought from another hospital and was given a 5 day course of it  Immunoglobulins treatment was started 4 days of being in hospital while still waiting for confirmation of test results  The Immunoglobulins treatment helped, but it got worse and later improved as the doctors suggested  Treatment with a course of Immunoglobulins helped to start getting better after a few days in hospital  Treatment in hospital was mainly with Immunoglobulins support with physio and OT  Was given Pregabalin for pain in hospital  Was treated with Immunoglobulins after diagnosis of GBS  Was treated with plasma exchange and immunoglobulins  Used to have eye drops for eyes  Used to manage pain on feet with lidocaine patches  Was eventually given Plasma Exchange, antibiotics and steroids having already received Immunoglobulins  Was in a coma but can remember Immunoglobulin was given  There was some improvement a month after the Immunoglobulins treatment  The consultant knew what to do and got the Immunoglobulins started on the first day, which helped to stop it worsening  Neurologist said was optimistic of a good recovery after immunoglobulin treatment  Had pain killers and immunoglobulins or plasma exchange  Unlike other variants, plasma exchange is not treatment of choice for AMAN variant  Initially had a course of Immunoglobulins which didn't work, so a course of Plasma Exchange was added  You have to keep going over a long term with physiotherapy immunoglobulin as required |
|  | Lack of follow up care | A long term follow up by consultants will be helpful, as there is currently not enough follow up  After discharge from hospital, it was like being eventually left to manage on your own  Feeling like giving up because of lack of follow up  Inadequate recognition of residual problems  Need for follow up  Neurologists should have a yearly review of GBS patients becuase of the residual effects etc  One concern is the NHS hasn't got after care for people with GBS which doesn't help with the residual symptoms  Programme to see people after discharge over a long term is best because you recover from GBS slowly  The absence of after care in the NHS for GBS patients leaves some patients anxious about the future health and wellbeing  Felt there was no adequate support  Backup or follow up care from NHS was very poor and it links with the lack of knowledge on GBS and other rare conditions  Difficulty in follow up care due to failure in communication between services (primary, secondary and geographical)  Was not offered physiotherapy but has made self-effort towards recovery and independence  Felt like the system has let him down  Health care follow up plan  It feels like there is no support available and you just have to cope wih it  All support including OT and physio should be offered for long term  Had a lot of physio exercises after discharge from hospital and movement is now quite good  Hydrotherapy is available in the hospital but can't access due to low staffing levels  Initially saw physios at home twice a week dropping to once a week as condition improved, then stopped seeing them  Physiotherapy immediately after discharge would have been helpful for building confidence in walking and balance  All neuro conditions should be grouped together for the purpose of follow up care because they are all neurological  The GP could invite GBS patients for yearly check up  It will be helpful to continue with physio at home and hospital as already in place |
|  | Support with ADL | Carers help with personal hygiene needs while wife and daughter are out to work and college respectively  OT helped with ADL  Feeling annoyed with help offered because of youth  OT and speech therapist were very good in reassuring and supporting with ADL  OT helped with arranging grips over bath  Physical aids provided by OT at home  Inadequate rehab resources unless you can pay  The gym which has now closed and had to resort to exercising at home  A special rope is installed at home to help with access to toilet upstairs  Able to feed and do personal care, but has sometimes helped because fine motor skills are not very good  Was able to return home after council put a second handrail to go upstairs  After rehab, could walk with stick and dress up but with a bit of difficulty  At rehab, moved from a big reclining chair to a wheelchair, then to a frame for a few days before sticks  Attending a gym with help from friends and family has enabled progress  Takes wife 3 hours to get me downstairs at which point I can make breakfast  Can walk short distances around the house but has to use folding down motor scooter to go to the supermarket  Currently using hoist and sleeping on a hospital bed at home, which is unusual  Stayed downstairs and used commode because couldn't go upstairs initially after leaving hospital  Uses sticks to walk at home because of lack of balance  Uses walking stick to help with balance  All the leisure facilities in the UK should be funded by the local councils and made accessible to all, but the austerity situation makes that difficult |
|  | **Disease factors** | |
|  | Initial or early physical problems | Also had problems with eyes such as white flashes and watering because of GBS  Another barrier is extreme pain in the legs with red hot pins and needles especially at night  Believes illness related to food poisoning as had eaten heated lamb prior to developing initial symptoms of the illness  Can't control legs because brain isn't in contact with my legs due to the GBS  Could write only with assistance at the time of discharge from hospital  Couldn't do much initially following discharge because of grip problems, but family helped while improvement eventually came  Couldn't feel my legs when it started and using the Tube was difficult  Couldn't move right leg while on holiday  Difficult to lift and open things but husband and son help  Drops a lot of things due to reduced grip especially with small things  Easily falls over at home while trying to walk at home because legs are weak  Everything takes longer and tires out quicker  Eye sight went double in the 3 months leading to diagnosis of GBS  Felt like a 1 year old baby when first started exercising at rehab  Felt pain in right leg and foot while at work, which worsened and GP referred to hospital with GBS in mind  Felt really ill, with weakness in arms, legs then became more of paralysis when it happened  Felt unwell a day after chilling out and thought it was tiredness  Felt unwell and went to see Neurologist and nerve conduction test showed only residual damage to nerve sheath but not new GBS  Felt unwell six years ago and GP suspected GBS and referred to hospital  Felt well looked after with the first episode of GBS, unlike this second time |
|  | Residual or late physical problems | Can't work and in wheelchair a lot of the time  Difficult to get into the pool, but there usually help from people to get in using a hoist  Evidence suggests many people with GBS cannot function at work after up to 20 years of getting GBS  Fatigue is a common complaint among GBS sufferers on Facebook  Fatigue remains a problem but work and family have been supportive  Feels was given immunoglobulins treatment much later  Got stronger and stronger in the first 2 or 3 years  Had to learn how to hold things, brush teeth, hair etc  Had to learn how to write again after coming out of hospital, but it came back quick  Had to stop all the medicines except one for BP, because of the psychological problems experienced with cocktail of medicines  Hardest barrier to recovery is the difficulty in balancing  Has been able to wear only pyjamas because of massive oedema caused by GBS  Has had 7 years taken out of life but different people recover differently  Has made good recovery but still has residual effects like many others  Has made pretty good recovery but still has residual symptoms of considerable weakness, tingling and fatigue  Has made up to 95% recovery but still residual problems of pain and mobility issues  Has not been able to wear shoes because legs swell a lot  Has not made full recovery due to persistent residual symptoms  Has oedema in legs due to GBS attacking legs  Has recovered except residual symptoms of fatigue and partial facial paralysis  Has residual symptoms of fatigue and partial face paralysis  Has recovered up to using crutches at home and wheelchair outdoors, which is progress as compared to being paralysed in hospital  Has some residual symptoms but not as bad as other read about  Has to wait 26 weeks to see neuro specialist for itching because the neuro specialists are not many  Has tried to manage the stigma by either not going out or hiding the crutches from people where possible  Has very little recovery since hospital  Have adjusted well but had to reorganise as can't do things as before  Have not yet recovered fully as still experience numbness and pain  Have read about breaking teeth and eye problems over the years  Have recovered reasonable well but residual effects hampers full recovery  Have seen stuff about teeth, eyesight and memory but not sure if there is more to it  Haven't slept well since getting GBS because of discomfort - e.g pain in back of legs  He did have one moment in the hospital where he wished he could just go  Heard about long terms effects of GBS on teeth and eyesight, but there's need to know  All physical functions have returned to normal except feet and toes being a bit dodgy with pain |
|  | Comorbidity | Got pneumonia while on holiday and was taken to hospital  Had Autoimmune Haemolytic Anaemia which made it more difficult to treat the GBS  Recently out of hospital due to kidney stones following GBS  They found I had other complications because of GBS  Was born deaf and wears hearing aids  Wife says husband already has vitiligo, another autoimmune condition as GBS  Had low BP alongside GBS and felt very weak |
|  | Sequelae or complications | Bladder hasn't returned to normal since having catheter in hospital due to GBS  They found I had other complications because of GBS  Has heterotopic ossification as a complication of the GBS illness  Got colonised with candida auras while in hospital  Feels the weight gain is due to lack of exercise, depression and alcohol intake |
|  | Problems due to treatment of GBS | The osteoporosis was a result of the drugs  Had an allergic reaction, but can't recall whether it was during or after the Immunoglobulins treatment  Developed ankle fractures due to intensive exercises and osteoporosis |
|  | Being young or fit prior to GBS is helpful | Younger age helped with recovery  Being fit before GBS probably helped with recovery  Being young helped mentally when it happened  Was reassured that being young combined with correct treatment could speed up recovery |
|  | Self-management | Advice from one GBS patient to another - keeping with physiotherapy and exercise, get involved in some activity to take mind off and be positive about recovery  Gym for disabled people was brilliant - NHS should support that  Patient led Hyperbaric chamber- which seems to help with nerve damage, and people with MS already use it |
|  | Independent or self-effort | Have started doing stand-up comedy to increase awareness  Did own house assessment because it was taking too long to get out of rehab unit  While in hospital, used to mobilise around to avoid muscles wasting  Has to be actively doing something - mentality of looking forward  Although struggling, still tries to keep busy with house chores rather than laid flat in bed  Found a nearby gym, an extremely helpful coping strategy  Life has changed - not working anymore but trying to help in the house as much as possible  There are difficulties, but you either find a new way of doing things or get help from someone and carry on as far as possible  Advice to others - work past physical limitations and stay positive  Learnt to use Internet for shopping - adapting to being house bound  Uses a blanket cradle to keep duvet off feet and reduce pain  Wants to try CBD oil to see if it will help with the pins and needles  Exercise has helped in progressing from being paralysed to using crutches to walk  GP practice runs a course called Active Lincolnshire which can be helpful  Important to maintain a good immune system because low immunity is a problem with GBS  Has been taking vitamins but has not been advised by a doctor to do so  Taken up palates to help myself  Discharged from hospital with physiotherapy and gym 3 times a week, then swimming twice a week |
|  | Remaining active is helpful | Another recovery strategy is to maintain activity e.g swimming, gym, physiotherapy etc  It is important to do what you are told to do, if possible swimming, is very helpful  Use of a pool in a nearby leisure club has been helpful  The hydrotherapy using the pool is the best because the surrounding water supports and gets your legs used walking motion  Has been advising people on GBS website to use the pool to help with balance and standing up  staying active can take your mind off GBS |
|  | **Psychological factors** | |
|  | Being positive and determined to get well | A positive mental attitude helped with recovery, or one will suffer terribly  A positive message when conveyed by consultants or neurologists will help with recovery  Advice for others -keep going and keep a diary if possible  Advice to help others includes - being positive, discussing with employer, be as normal as before etc  Always be positive even if you don't make full recovery  An incredibly positive Buddhist person  Being a positive and lucky person helped to stay normal  Being positive, wife and family and treatment in hospital helped with recovery  Believes with hard work can get back to playing volleyball at national level as before  Determination and being positive to get well have helped with recovery  Determination and faith helped with recovery  Determination and push can help  Determination to get well and be back playing valley was a driving force back to normal activities  Don't give up and ask for help if necessary, that should help  Don't give up and be determined to do things and recover  Don't give up, keep trying  Getting GBS the first time and recovering was a motivation that it is possible to get well again  Has been a doer before GBS and just can't stand around doing nothing  Has been positive and telling friends that will get better after a while rather than saying long term sick  Has got no worries about things that can't be changed |
|  | Feeling lucky as compared to others | At age of 69 , will be grateful to recover beyond using elbow crutches  Feels fortunate as compared to other people who are on their own and struggling  Feels glad about current level of recovery as compared to earlier state of paralysis  Feels has recovered well as compared to others  Feels lucky for not being paralysed  Feels lucky to have recovered well, but still has residual problems  Feels lucky with many things but some things can anger as they can be controlled such as sleeping too much  Felt doing well recovery wise as compared with others on GAIN forum  Felt lucky as almost faced death  Felt lucky to have two daughters who helped to sort things out, it could be different without someone to help  Happy with still being here to offer advice to others with GBS  Has made a good recovery and is proud of that  Has made almost complete recovery  Has made some good recovery sooner than expected  Other people can be in worse situations |
|  | Seeing current situation as an opportunity  for other options | Will advise anyone with GBS not to give up and take that change as an opportunity  Take that change as an opportunity to do something you always wanted to do  A crisis is also an opportunity  Change is not always bad, it can be good  Has taken up old hobby of fishing and DIY house decorating etc which have been helpful |
|  | Moving from hospital to home environment | You can recover better in your own environment but it's better not to overdo things  Staying at home and accessing care in hospital has been helpful  Stayed at home and got better the first time of getting GBS  Getting discharged and staying at home helped with recovery  Had enough of staying in hospital after 2 weeks in acute ward and 2 weeks in rehab ward |
|  | Use of psychological aids | Husband brought a jigsaw which was helpful with coordination and concentration  Listening to favourite music has been helpful  Neurologist helped with scrambled memory and getting brain into the right place |
|  | Having or intending to have counselling | Someone visited at home to talk about any psychological issues  Went back to GP after discharge from hospital and requested for psychological support  Had counselling for PTSD after recurrence of GBS in 2013  People with GBS or CIDP should be offered psychological support because it's a life changing condition  Group sessions for people with the same GBS condition will help with psychological issues  Background in psychology helped a bit  Counselling would have been helpful but didn't have the opportunity  Needs to talk to GP about counselling because of frustrations sometimes  Currently has an appointment for PTSD due to going from being manager of a big company and travelling around the world to nothing since getting GBS  Strong background has helped to cope with GBS when it came |
|  | Adjusting or changing life activities | Adjusted to normal like by reapplying for driving licence and starting to drive again  Avoiding stressful situations also helped with coping  Building stamina and knowing my limits helped with return to work and routine activities  Has not had cannabis since GBS and does not smoke cigarettes  Have accepted that having short naps helps with overall day  Have adjusted by planning ahead, sleeping early and eating well  In terms of adjustment, have tried mentally and physically to do things but wife still supports  In the hospital, was so please to be able to move and take a few step across the ward, but with caution  Just have to change your way of life  Managed to slowly do things as was mostly at home alone  People recovery from GBS should also try to get back and start doing the things they did before as much as possible  Setting small goals and keeping that in mind helped a lot  Started to do things on own as gradually improved  Was a bit selfish in setting boundaries that didn't favour family members  Advice to others include -1. Setting small goals, 2. Keep a diary, 3. Appreciate your achievement, 4. Don't worry about the loss, 5. Think yourself lucky |
|  | When given useful or reassuring information about GBS | Was told people are different as some recover fully while others don't  Was told at the hospital that recovery will take up to 6 years, so has resigned to the fact that it's not a quick fix  Was reassured that being young could help with full recovery together with the Immunoglobulins treatment  The anxiety disappeared at the hospital once the test showed the GBS had not reached the lungs |
|  | **Social factors** | |
|  | Positive impact of GBS and associated conditions on family members | Impact on daughter who has anxiety and depression has been positive as she has adapted to going out without me because my illness |
|  | Attending or interacting at faith based meetings | Coped through Buddhist meetings and reading stuff  Coped by relying on faith and sharing such faith and beliefs with others  Attending Buddhist meetings also helped with coping |
|  | Sharing experience with others as a GBS patient | A volunteer GB patient visiting hospital and interacting with patients will be good  Sharing experience by speaking to others as a volunteer previous |
|  | Supportive family and friends | Also had support from someone in opening lids etc in the kitchen  Another key help was a work colleague having it two years earlier  Children and family members were surprised or even worried when they got to know after about 3 months in hospital with GBS  Everyone was helpful, but no one knew about it  Family and determination to regain independence helped with recovery  Family and friends have remained supportive  Family has helped a lot in the recovery journey  Family, especially children have been supportive  Friends visited in hospital to show support  Had support from family after discharge  Having access to people who have had similar experiences will be helpful  Having someone to talk to and share the experience has been helpful  Initially after leaving hospital mother helped with cooking and husband helping with showering  It was helpful to have a network of strong people around, family and friends  It would have been difficult without family support  Lives with husband  mum was very helpful in supporting and providing appropriate care  One of the best movements was when helped by friend to pick my son in school on a wheelchair and later using a Zimmer frame  Partner helps with balance and walking when going out together  People might get a bit surprised the first time they see you in wheelchair but they accept you for what you are  People outside there are quite understanding and supportive |
|  | Interaction with others on GAIN site and Facebook | Everyone's journey is different but it would help to speak to someone who had experienced it  If it were not for social media, fewer people would know about GBS  GAIN is a good forum because you can talk to other people with GBS all over the world and get information  Information on Facebook groups is helpful but there are no answers about recovery as everyone is different  Speaking with others who have had GBS could help  People need to find what they are interested in and join a group that keeps them mentally and socially active  Advice for others - make use of Facebook and other online support networks  Online peer support  Advice for others - engage with physio as much as possible and ask somebody |
|  | Supported by social care and others | Had to notify DVLA while in hospital, now able to drive on medical review licence due to the condition  Had an au-pair to look after children during first episode of GBS  Had carer support during first 2 weeks after discharge in the day, but husband helped in the evenings  Will need social care support to be more independent  Uses wheelchair to mobilise that was paid by GAIN |
|  | Ongoing struggle for disability facilities | Currently fighting for disability facilities at work side, which is a problem  Currently in a legal dialogue with the Spa gym, because of lack to lift to access gym upstairs |
|  | Resources are kept away from people who need them | No help out there for children of disabled parents  So many people are abandoned with GBS and assuming they have recovered and back to normal  The resources are taken away from people who really need them  The panel dismisses GBS patients seeking benefits because they can't see the disability  The work Occupational Health don't agree that the illness is a disability  The residual effects are not recognised as disability and they should be  People can't see GBS because it is with the nervous system  People stare and shout abuse because you don't look you have a disability  Some GBS patients have difficulty accessing benefits like the Universal Credit  Unable to find support for son of a disabled person  Fell out with GAIN because many people there just want to moan and I am not moaner  Social care service can help people with GBS to get benefits like Personal Independent Payment (PIP), blue badges etc  Social services could help with long term physiotherapy for GBS patients  Social and health services could also advise GBS patients about the support provided by GAIN |
|  | Disturbed marriage or partner relationship | Girlfriend helped to get back to health but left as life had changed which wasn't fair for her  Marital relationships are not doable at the moment  The illness is reason for getting divorced |
|  | Stigma in being seen as disable | Didn't want to socialise to avoid being seen in wheelchair by friends as had been a very active person  Doesn't usually socialise with friends but goes out with family  Has been selective in terms of where to go because of stigma associated with using wheelchair or crutches  One barrier to recovery was pride, not wanting to be labelled as disabled  People who have known you before, seeing you in wheelchair or using crutches is very stigmatising and embarrassing  Was concerned about being in wheelchair for rest of life but felt better after immunoglobulin  Not wanting to be seen in a wheelchair was a very big psychological barrier |
|  | Undue burden on wife or partner | The impact of the illness on wife has been negative as she has over works herself looking after me  The impact of the GBS was stressful and panicky for wife as she had to run our home for the first time, with everything not in her name |
|  | Social life is at stand-still | It is frustrating when people don't help around the house as can't do everything  Life has turned outside down with the GBS  Life is now completely different from before  Now forced to depend on other people  Part of the impact of this illness is being stuck in a small house instead of the big house we sold because of difficulty in climbing the stairs  Socially life came to a complete standstill  Socially, went from being a steely manager in a big company to getting GBS which changed everything  When you are in hospital you get more visitors but people are more distant once you feel better  You miss out on what your family and friends are doing when you are ill |
|  | Negative impact on family members | Family and partner were told recovery might never happen or it might take a year by limb if it did  Family doesn't understand that residual symptoms of pain persists for long  Family were terrified because they were told bad news at the hospital at the start  Felt like the system has let them down quite badly  Impact on parents was bad as they are old and poorly, and found it difficult but won't talk about it  It probably affected mum more than any one  Now has a different partner who is understanding  One of saddest things was being in coma and missing son's birthday  Parents (mum and dad) were very scared because they have always regarded me as successful person  Has been a tough time for family as both children have disability adding to mom's GBS  The GBS has affected personal income, but family is comfortable because of wife fabulous retirement package  Has tried to protect children by acting normal |
|  | Difficult with child and home care | It was difficult doing things with five year old son at home  It was difficult getting five year old son to school but other mums helped while husband was at work  Partner has a son, and it's difficult to run around after a 4 year old  Partner helps but has to drive because partner can't drive  Son is upset because family moved home from South to North  Used to be very much involved in son's education  Would love to do more for son's education but can't get out much  Constantly worried that son is missing out on activities |
|  | **Occupational factors** | |
|  | Supported with work related benefits | Moved from 50k a year job to benefits but no regret  Also had a taxi grant from Department of Works and Pensions to travel to work  DWP has helped with grant for Access to Work, which helps with back and forth to work  No financial problem currently because getting Employment Support Allowance  It will be interesting to find out how many people with GBS are no longer working or doing stuff after 20 years |
|  | Supportive employer and work colleagues | Boyfriend's employer was also supportive as they allowed him to time to stay with  Employer has been flexible in terms of hours of work with no change in salary  Employer has been helpful with work schedule with no change in salary after 9 months of staying away with GBS  Employer has been supportive but they have to respect the civil service rules  Employer was also very helping in visiting hospital to offer encouragement  Employer was supportive with workload and schedule  Line manager and work colleagues have shown understanding and support in and out of hospital  No financial problems because employer continued paying salary  Returned to work after a year post discharge from hospital  Returned to work after the long sickness off work, and agreed with occupation health to a 50% work hours  Returned to work on reduced hours because of vision problems but that has cleared now  Some teachers supported by visiting in hospital  Still not fully recovered fully but work has been very supportive  They were very understanding in my last contract job  Was off work for 9 months after leaving hospital 6 years ago  Was paid full salary throughout 9 months of absence from work due to GBS  Went back to work on reduced hours  Work is normal and no one knows I had it unless I tell them  Worked as assistant child psychologist and back to the same job after GBS  Worked in office initial upon return to work but now back to the same role as before  Works from home on Wednesdays to help with fatigue |
|  | Financial burden due to not working | The negative financial impact is significant as currently on benefits which is nothing compared to previous salary of £213, 000 per year  The lack of income means that we can't go to a lot of places  Motivated to return to work and other activities in order to make money  Many people with GBS could be facing financial difficulties  It has been a massive financial hit, but managing with wife's teaching income  Impact on work was negative as was not the same person again and employer was not very supportive  Financially it has been difficult but manageable |
|  | Forced to change current and future work or school schedule | Planning on doing a teaching course  Missed the second year of my A 'levels' which wasn't good  It changed my choice of university and the course  Returned to college but could not do fulltime initially  Has returned to normal hours but restricted to work in only parts of the Prison  Have adjusted to working part-time due to illness and son's disability  Still does part time tutoring which helps |
|  | Forced to take early retirement | Currently being urged by employer to take ill health retirement, as the job needs to be done  Gave up working with governors of a school due to getting Miller Fisher the second time  Had early retirement from work due to GBS  Left paid employment but did secretary work for GAIN for 5 years after getting GBS  Work suffered because memory was bad  Feels will be better off financially with a dismissal option than ill health retirement  Was self-employed but has not worked properly since getting GBS  The GBS and its recurrence affected role in teaching PE, ending up in early retirement |
|  | Looking for a suitable job as unable to do old job | Has stopped old job due to residual symptoms  Looking for a suitable desk based job but it's difficult to find  Hate being unemployed as has worked so hard to get to a high powered job  In my last job, lunch time was a choice between eating and sleeping  Can't get a job because of chronic fatigue and difficulty in walking  Has stopped old job due to residual symptoms  Had to give up work after 10 years because of chronic fatigue and could not cope  Currently having issues with job because of the illness situation  Had stopped teaching before GBS but went back to teaching after firs episode of GBS |
| The importance of communication and appropriate information sharing for recovery | Communication problems (voice not heard or understood)  Felt not listened to | Felt frustrated because GPs were not listening and wouldn't appreciate that it was more than diazepam and rest  Felt not listened to by experts in the hospital  Wanted to go home and they said should stay there with no explanation about condition |
|  | Speech difficulty | It was difficult to communicate as unable to speak  Communication is difficult when you can't speak  It is difficult and frustrating if you can't speak  There was a lot of frustration when it was difficult to communicate, but use of visual support and speech and language helped |
|  | Support with GBS information | GAIN is very informative but most people are there just to moan  GAIN is a little community where a lot of information is shared which benefits those who don't know  GAIN literature was the first indication that I would get well  GAIN literature was good because it indicated many people make full recovery  GAIN website and Facebook were also helpful in knowing you are not alone in terms of GBS symptoms, length of stay in hospital etc  GAIN website and Facebook, as well as reading other GBS sufferers' stories has been helpful  Got information about GBS from NHS website, GAIN Facebook  Had information from GAIN on the internet and was very keen to understand more  Husband printed whole lot of stuff from GAIN website  Information about Miller Fisher was found by Googling and reading about it  Information form Guillain-Barre Society was helpful with driving and licence arrangements  A work colleague helped with reading of GAIN information left by husband  Looked for information online with wife  Partner researched and Mum collected more information on GBS from her GP  Members of the GBS Facebook are from all over the world  Like in stroke cases, volunteers who had it could speak to people who have it  There is a need for some sort of support network for these people  Found out from interaction with other people with GBS that children can spread germs easily, which is not good with GBS |
|  | Information provided by health care professionals | Some care staff in the hospital also helped with information on GBS and GAIN  Uncertainty about the future  Was told about need for regular physio and that everything will return but will take time |
|  | None or limited information given to patient about GBS | Consultants explained the extent of damage but not certain about recovery or relapses  Contrarily to what many people say, you can get GBS again  Consultants said a lot but not exactly where it was heading to  Had been told after first episode of GBS that it might not recur  It is very worrying when you are paralysed and the people don't know if you will get well  It was difficult that some of the doctors at the beginning didn't know what was going on  No information from hospital, only GAIN  No information given or was not explained to patient's understanding  Nobody explained to me about GBS because nobody knew much about it  Not knowing what is going to happen is emotionally upsetting  Not much information was received from hospital staff about GBS  The hospital didn't give much information, and had to look it up online with support of wife  They didn't know what to do because there was a relapse after each 5 day treatment session  Was given only very brief description about GBS in hospital, but googled for information on internet after discharge from hospital  Was not told anything about what would happen  Was not told anything at the beginning as they didn't know what was going on  Was not told much about recovery before leaving hospital  Was not told what it was or what would happen  Was very keen to learn more about it from other people on GAIN  Had never heard of GBS and no one could say whether you would get well |
| Awareness, knowledge and information provision | Knowledge gap among health care providers | The nurses didn't know about GBS  Student doctors wanted to learn about it  They didn't seem to know what it was  Many doctors came to see me and to learn about it  Was the first GBS patient that the physio centre ever had  One problem was a lot of the nurses and doctors didn't know about GBS and the need to physiotherapy early  Not all the nurses and doctors knew about GBS but that's because it's rare  The nurses need training because they didn't know how to care for a GBS patient  The residual symptoms should be recognised and taken seriously  There isn't much information from NHS  More information on the residual effects will be helpful  Good to have NHS champions on GBS to provide information or advice rather than a Charity providing information  Suggestion - as part of future research, look at effects of GBS after 5 years, 10 years or 20 years, where people actually are rather than what the book says  Local GPs don't know that GBS is complex and the need for backup support  Lack of awareness as with other rare conditions  Like other rare conditions, there is not enough awareness among GPs about GBS  Will need more information to understand more about the facial paralysis and other residual effects  Doctor who confirmed the diagnosis came from Denmark because the doctors in Portsmouth then didn't know much about GBS  Hospital staff still don't know much about GBS  The problem was no one knows that it was GBS |
|  | Lack of public awareness about GBS | More people need to know about GBS  Leaflets could help with aware of GBS  It is the awareness about GBS that needs to happen  Need to raise awareness and encourage people to seek medical health as soon as possible if they feel ill  People stare and shout abuse because you don't look you have a disability |
| Redefining recovery | Recovery milestone | It is quite and exciting achievement to do little things  One of the best moments was being to hold a phone in the hospital  Was able to get upstairs on own 2 months after discharge  Was expected to walk with Zimmer frame and climb steps, so managed one or two steps and got discharged |
|  | Redefining recovery as an individual and accepting that | Meaning of recovery is relative to the individual  Being unable to do routine things like putting own underpants was really upsetting although short-lived  Being able to hold wife's hand while walking means getting back to normal  Accept that recovery could be slow and people are different |
