# Supplementary material for: Exploring the experiences of having Guillain‐Barré Syndrome: A qualitative interview study
Source: Health Expect. 2020 Aug 3;23(5):1338–49. doi: 10.1111/hex.13116 (PMC7696117; doi:10.1111/hex.13116)
Supplement: Supplementary file 3 — Table S3 [file HEX-23-1338-s003.docx]

| No | **Themes** | | | **Subthemes** | **Quotes** | |
| --- | --- | --- | --- | --- | --- | --- |
| 1 | **Importance of early diagnosis** | | | Early detection/delayed diagnosis | “We went down to the doctors. He examined me and that was when he  recommended I go straight to hospital; which I did and was admitted;  and from day 1 they thought it was GBS.” (P5).  “They didn’t find the GBS until I had the nerve conduction study. That  was twomonths after the holiday.” (P3). | |
|  |  |  |  | Misattributing symptoms | “So we got to hospital. They were doing lots of different test. First they  thought it was meningitis or MS” (P2). | |
| 2 | **Experience of inpatient care** | | | Delayed treatment | “….I think if I had had that treatment earlier, it would have been better”  (P5). | |
|  |  |  |  | Positive/negative experience of care | “In the intensive care unit, there was a wonderful nurse. She was  absolutely fantastic because I have got long hair, she would help me  wash my hair; she would braid my hair to stop in getting tangled  because I can just move my head a little bit: the back of my hair was  getting tangled. She would wash and braid my hair. She would spend the  most time with me to try and lip read me and spend time with me; but  some of the other nurses where not as nice at all; very unpleasant.” (P1). | |
| No | | Themes | Subthemes | | | Quotes |
| 3 | | **Support for recovery** | **Healthcare factors that help or hinder recovery e.g** | | | |
|  |  |  | *Early/appropriate investigations* | | | “Yes, I went to the neurologist, and he saw me in the afternoon and I was admitted to the hospital. I got nerve conduction tests and was admitted to the hospital; had a lumber puncture, and put on immunoglobulin that night.” (P4). |
|  |  |  | *Adverse effects of treatment* | | | “I can’t recall if it was when I was having my treatment or straight after,  but I started getting an allergic reactionon my hands.” (P15). |
|  |  |  | **Disease factors that help or hinder recovery e.g** | | | |
|  |  |  | *Age, activity* | | | “Really drive myself; before I was ill, I was running 10 miles and things like that. The other thing that probably helped with the recovery, when I was taken ill with it, I was really fit. That may help. I was running, doing spin classes; really fit. You wouldn’t think so to see me now!” (P7).  “I think being young helps your mentality when it happened: you have amore positive outlook on life.” (P2). |
|  |  |  | *Residual or late physical problems* | | | “Not fantastic. I haven’t made a full recovery. I still have peripheral neuropathy in my legs and hands; and my memory is just shot. I suffer with chronic fatigue as well.” (P1) |
|  |  |  | **Psychological factors that helped/hindered recovery** | | | |
|  |  |  | *Being positive* | | | “Positive mental attitude. It is all about attitude: if your glass is always half empty, you are going to suffer terribly.” (P3) |
|  |  |  | *Initial psychological problems/residual psychological effects* | | | “I had dark days where I thought I don’t want to live if I am going to be  a burden to my family; if I am going to be paralysed, I don’t want to be  here.” (P10).  “I still get night terrors. Basically my partner just has to hold me; he  can’t wake me up from them; and I am screaming in my sleep. So I still  get them.” (P1) |
|  |  |  | **Social factors that help or hinder recovery** | | | |
|  |  |  | *Sharing experience with others* | | | …”and I think you need role model as well. I now volunteer through the  GAIN charity. I go into hospitalsand I visit people.” (P6). |
|  |  |  | *Stigma of disability* | | | “I think the one big problem I did have, I suppose, was my own pride. I  didn’t like to be seen as disabled. I didn’t like that label put on me, but  unfortunately that is how I was labelled, and to a certain extent,  possibly seen to be even now, but I just didn’t like that disabled label.”  (P4) |
|  |  |  | **Occupational factors that help or hinder recovery** | | | |
|  |  |  | *Change in work schedule, job (role), retirement* | | | “I haven’t been to work ever since it happened. I was working for  myself.” (P3).  “I went part time at work after GBS. There are days when I can’t work,  or my hands won’t work. There are days when nothing works; you have  good days and bad.” (P10) |
|  |  |  | *Supportive employer and/or work colleagues* | | | “My company were extremely supportive. They gave me little bits of  work back at a time. I wasn’t stressed or loaded on. Most Fridays and  Mondays, I try and work from home. So I have a long weekend. I  haven’t got to travel or go out of the house. Then I go on Tuesdays,  Wednesdays and Thursdays and know I have four days of sitting at  home if I need to. That is still the case at the moment.” (P11). |

| No | Themes | Subthemes | Quotes |
| --- | --- | --- | --- |
| 4 | **Communication** |  | “Nothing. I wasn’t told anything. I wasn’t told what I had or what was going to happen. I wanted to go home, and they said I should stay there; but they didn’t tell me why or what I had or what was going to happen; at the beginning. It wasn’t until I was on a tracheostomy, and the consultant came in, and because I couldn’t speak, the nurse was lip reading me, and I asked what do I have? I remember Dr Steve, and he said –‘Oh you are really lucky, you have GBS!’ I thought I’ve never  heard of GBS. That is when I found out what I had, but I still had absolutely no idea what that was.” (P1). |
| 5 | **Awareness, knowledge and information provision** |  | “It is the awareness that needs to happen. I see about it everyday because I follow it all on Facebook. I am in many different groups; so you see people talking about it every day, but if there weren’t any social media, even less people would know about it. If it wasn’t for social media I wouldn’t have met A and gone to see his production. In 14 years, he’s the only one I have met who has GB.” (P2). |
| 6 | **Achieving the ‘new’ normal** | *Adjusting or changing daily activities* | “You have to adjust mentally and physically because there are things that I can’t do that I have to get other people to help me with. Even to this point now the wife will say she’ll help me to get dressed. I want to do them myself but there are things I can’t do like getting my underpants on etc. I’ve no chance; she has to help me with those. I liken it to the soldiers who have come back from Afghanistan who have had their legs blown off. They can look down and they have no legs, so it is going to affect them mentally but they always get up and continue with life. They still have a life, but it is a different life.” (P3). |
|  |  | *Redefining recovery* | “Yeah, good. I have adjusted pretty well I think. It is getting back to as near normal as possible; apart from the physical side. As an example, the weather here this week has been beautiful. Sunday, my wife and I went down to the village – it was nice; because I had got my electric wheelchair, I was able to hold her hand while we walked down to the village. That to me is getting back to normal. It shows normality is returning.” (P13). |

**Table S3 Details of themes and quotes**
